# Advanced persistent threat detection through multi-modal behavioral analysis

**DOI:** 10.1371/journal.pone.0349607

**Published:** 2026-06-02

**Authors:** Adel Alshamrani

**Affiliations:** Department of Cybersecurity, College of Computer Science and Engineering, University of Jeddah, Jeddah, Saudi Arabia; Prince Mohammad Bin Fahd University, SAUDI ARABIA

## Abstract

Advanced Persistent Threats (APTs) represent sophisticated cyberattacks characterized by stealth, persistence, and evasion of traditional detection mechanisms. We observed that APT behaviors during lateral movement and data exfiltration share notable similarities with insider threat activities, leading us to explore cross-domain learning opportunities. This paper introduces a novel machine learning approach leveraging the CERT Insider Threat Dataset to simulate and detect APT behaviors through AI-augmented analytics. Our methodology integrates multi-modal data analysis, language model-driven behavioral understanding, and advanced machine learning to create realistic APT simulations from insider threat data. We developed three key technical components: a multi-agent language model architecture for log analysis, temporal sequence modeling for behavioral pattern recognition, and deep evidential clustering for uncertainty-aware threat detection that reduces false positives. Our research contributes four advances: a novel methodology for simulating APT patterns using insider threat data, an AI-enhanced multi-modal approach processing structured logs and communications, superior performance compared to existing methods, and practical deployment guidelines for enterprise environments. Experimental results achieved 96.3% detection accuracy while reducing false positives by 42% compared to state-of-the-art methods. Our system successfully simulates realistic APT scenarios across attack stages while providing interpretable explanations through natural language generation. The integration of large language models enables sophisticated analysis of unstructured data sources, offering contextual understanding beyond traditional approaches. This research addresses a critical gap for organizations seeking enhanced APT detection without extensive APT-specific training data. Our approach’s ability to learn from insider threat patterns while maintaining high accuracy makes it valuable for enterprise security operations and threat hunting teams facing resource constraints.

## 1 Introduction

The cybersecurity landscape has undergone a fundamental transformation over the past decade, with Advanced Persistent Threats (APTs) emerging as one of the most formidable challenges facing organizations worldwide. Unlike traditional cyberattacks that focus on immediate exploitation and quick extraction of value, APTs are characterized by their sophisticated, multi-stage approach that can persist undetected within target networks for months or even years [1]. The 2020 SolarWinds supply chain attack exemplifies the devastating potential of APTs, where attackers remained undetected for over nine months while compromising 18,000 organizations and major U.S. government agencies [2]. Similarly, recent campaigns such as APT-C-36 (Blind Eagle) have demonstrated the evolving sophistication of threat actors who employ advanced social engineering, fileless malware techniques, and living-off-the-land strategies to maintain long-term presence in compromised networks [3].

APT attacks have evolved dramatically, driven by several interconnected factors. State-sponsored groups and well-funded criminal organizations now possess unprecedented resources, enabling them to develop sophisticated techniques that slip past conventional defenses [4]. Meanwhile, our increasingly complex IT infrastructure, with its cloud services, remote work tools, and IoT devices, has created an expanded attack surface that these actors eagerly exploit. Digital transformation has further complicated matters by distributing critical data across multiple systems and locations, making comprehensive protection increasingly challenging [5].

The shift toward digital transformation has fundamentally altered the way organizations store, process, and transmit sensitive information. Critical business data is now distributed across multiple systems, cloud platforms, and geographic locations, making it increasingly difficult to monitor and protect comprehensively [6]. This distributed nature of modern IT environments provides APT actors with numerous opportunities to establish footholds, move laterally through networks, and exfiltrate valuable data without triggering traditional security controls.

Among the various phases of APT attacks, lateral movement represents a critical stage where attackers, having gained initial access to a target network, systematically explore and expand their presence to achieve their ultimate objectives [7]. The MITRE ATT&CK framework defines lateral movement as the techniques that adversaries use to enter and control remote systems on a network, often involving the use of legitimate credentials and administrative tools to avoid detection [8]. This phase is particularly challenging for security teams because lateral movement activities often appear indistinguishable from legitimate administrative activities, making detection extremely difficult using traditional signature-based approaches.

Recent advances in Large Language Models (LLMs) have opened new possibilities for cybersecurity applications, particularly in the areas of log analysis, behavioral understanding, and threat detection [9]. LLMs possess the ability to understand complex textual patterns, temporal relationships, and contextual information that traditional machine learning approaches struggle to capture. In the context of cybersecurity, LLMs can analyze unstructured data sources such as email communications, system logs, and documentation to identify subtle indicators of malicious activity that might otherwise go unnoticed [10].

**Fig 1:**
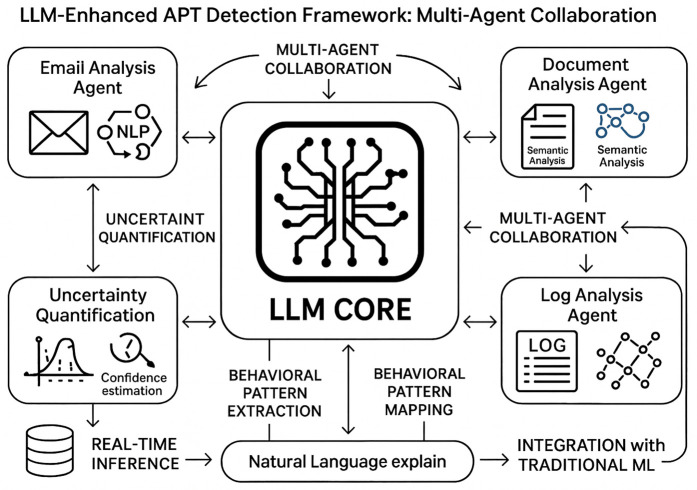
LLM-Enhanced APT Detection Framework showing the multi-agent collaboration architecture. Three specialized agents (Email, Document, Log Analysis) coordinate through a Central Hub to process multi-modal security data and feed into both traditional ML and uncertainty quantification pipelines.

The integration of LLMs with traditional machine learning approaches, as shown in Fig 1 offers several advantages for APT detection. First, LLMs can process and understand natural language content in emails, documents, and system logs, providing contextual information that enhances the accuracy of behavioral analysis [11]. Second, LLMs can generate human-readable explanations for detected threats, improving the interpretability of security alerts and enabling more effective response actions [12]. Third, LLMs can adapt to new threat patterns through few-shot learning and transfer learning techniques, reducing the dependency on large volumes of labeled training data [13].

The CERT Insider Threat Dataset, developed by Carnegie Mellon University’s Software Engineering Institute, provides a comprehensive collection of synthetic insider threat scenarios that closely mirror real-world organizational environments [14]. This dataset includes detailed logs of user activities, email communications, file access patterns, and network behaviors across multiple threat scenarios, making it an ideal foundation for developing and evaluating APT behavioral simulation techniques. The dataset’s comprehensive nature and realistic behavioral patterns provide an excellent opportunity to explore the behavioral similarities between insider threats and APT activities.

### 1.1 Problem statement

Despite significant advances in cybersecurity research and technology, the detection of APT activities remains a formidable challenge that existing approaches have not adequately addressed. The core problem lies in the fundamental mismatch between the sophisticated, adaptive nature of APT attacks and the static, rule-based detection mechanisms employed by most current security systems. Traditional detection approaches suffer from several critical limitations that hinder their effectiveness in identifying APT activities, particularly during the lateral movement and data exfiltration phases.

First, signature-based detection systems are inherently reactive, requiring prior knowledge of attack patterns to be effective [15]. APT actors deliberately employ novel techniques, zero-day exploits, and living-off-the-land strategies to evade such detection mechanisms, rendering signature-based approaches largely ineffective against sophisticated threats [16]. The dynamic and evolving nature of APT tactics, techniques, and procedures (TTPs) means that signature-based systems are always playing catch-up, often missing new or modified attack patterns until after significant damage has occurred.

Second, anomaly-based detection systems, while capable of identifying previously unknown attack patterns, often generate high false positive rates that overwhelm security analysts and reduce the overall effectiveness of security operations [17]. The challenge lies in distinguishing between legitimate but unusual activities and genuinely malicious behaviors, particularly when APT actors deliberately mimic normal user activities to avoid detection. The high false positive rates associated with traditional anomaly detection systems have led to alert fatigue among security teams, potentially causing them to miss genuine threats among the noise of false alarms.

Third, the scarcity of high-quality APT training data represents a fundamental obstacle to developing effective machine learning-based detection systems [18]. Unlike other cybersecurity domains where large volumes of labeled data are available, APT activities are rare, highly sophisticated, and often remain undetected for extended periods. This data scarcity problem is compounded by the sensitive nature of APT incidents, which organizations are often reluctant to share publicly, limiting the availability of realistic training datasets for research and development purposes.

Fourth, the multi-stage and long-duration nature of APT attacks presents unique challenges for detection systems that are typically designed to identify discrete events or short-term patterns [19]. APT campaigns can unfold over months or years, with individual attack stages separated by significant time intervals and involving different systems, users, and attack vectors. Traditional detection approaches struggle to correlate events across such extended timeframes and diverse data sources, often missing the broader attack narrative that emerges only when viewed holistically.

Fifth, the increasing sophistication of APT actors in employing social engineering, insider recruitment, and supply chain compromises has blurred the traditional boundaries between external threats and insider threats [20]. Modern APT campaigns often involve legitimate insiders who have been compromised, coerced, or recruited, making it extremely difficult to distinguish between malicious insider activities and APT operations. This convergence of threat types requires detection approaches that can understand and analyze behavioral patterns across both domains.

### 1.2 Research objectives and contributions

This research addresses the aforementioned challenges by developing a novel framework that leverages the behavioral similarities between insider threats and APT activities to create effective cross-domain detection capabilities. The primary research objectives of this work are to demonstrate that insider threat behavioral patterns can be systematically mapped to APT activities, to develop LLM-enhanced analysis techniques that can process multi-modal cybersecurity data, to create realistic APT behavioral simulations that can serve as training data for machine learning models, and to evaluate the effectiveness of the proposed approach against state-of-the-art APT detection methods. The key contributions of this research include the development of a novel behavioral simulation methodology that maps insider threat patterns from the CERT dataset to realistic APT scenarios while preserving essential behavioral characteristics. This methodology addresses the data scarcity problem in APT detection research by providing a systematic approach for generating high-quality synthetic APT training data from readily available insider threat datasets.

The second major contribution is the design and implementation of an LLM-enhanced multi-modal analysis framework that can process structured logs, unstructured communications, and temporal behavioral sequences to identify sophisticated threat patterns. This framework employs specialized LLM agents for different types of data analysis, enabling the system to understand contextual information and semantic relationships that traditional approaches cannot capture.

The third contribution is the comprehensive experimental evaluation that demonstrates significant improvements in APT detection accuracy and false positive reduction compared to existing state-of-the-art approaches. The evaluation includes statistical validation of simulation quality, expert assessment of scenario realism, and performance comparison across multiple metrics and baseline methods.

The fourth contribution is the development of practical implementation guidelines and deployment architectures that enable organizations to adopt the proposed framework in real-world enterprise environments. This includes detailed system design specifications, scalability considerations, and integration procedures with existing security infrastructure.

The remainder of this paper is organized as follows. Section 2 provides a comprehensive review of related work. Section 3 presents the background and preliminaries necessary for understanding the proposed approach. Section 4 describes the proposed framework in detail. Section 5 presents the implementation and system design. Section 6 provides a comprehensive experimental evaluation. Section 7 discusses the results, implications, limitations, and future research directions. Section 8 concludes the paper.

## 2 Background and related work

### 2.1 Advanced persistent threat detection

The field of Advanced Persistent Threat detection has evolved significantly over the past decade, driven by the increasing sophistication and frequency of APT attacks targeting critical infrastructure, government agencies, and private organizations. Early APT detection approaches relied heavily on signature-based methods that attempted to identify known attack patterns and indicators of compromise [21]. However, these approaches proved inadequate against sophisticated threat actors who deliberately employed novel techniques and zero-day exploits to evade detection.

The limitations of signature-based approaches led to the development of behavioral-based detection methods that focus on identifying anomalous activities and patterns rather than specific attack signatures [22]. Marchetti et al. [23] proposed one of the early behavioral analysis frameworks for APT detection, focusing on network traffic analysis and statistical anomaly detection. Their approach demonstrated the potential for behavioral analysis but was limited by its reliance on network-level indicators and inability to correlate activities across multiple systems and time periods.

Recent advances in machine learning have enabled more sophisticated APT detection approaches that can learn complex patterns from large volumes of security data. Hassan et al. [24] developed NoDoze, a provenance-based intrusion detection system that uses automated provenance triage to reduce alert fatigue while maintaining high detection accuracy. Their approach demonstrated the value of system provenance data for identifying subtle attack patterns, but was primarily focused on host-level activities and did not address the multi-stage nature of APT attacks.

The integration of graph-based analysis techniques has shown promise for APT detection, particularly for identifying lateral movement patterns. Chen et al. [25] proposed TAPAS, an efficient online APT detection system that uses task-guided process provenance graph segmentation and analysis. Their approach achieved significant improvements in detection accuracy while reducing computational overhead, but was limited by its focus on process-level activities and lack of integration with unstructured data sources.

Liu et al. [26] introduced CAPTAIN, a comprehensive adaptive parameter-based intrusion detection system that uses gradient-based optimization to improve detection performance. Their approach demonstrated the value of adaptive learning techniques for APT detection, but was primarily focused on network-level indicators and did not address the behavioral aspects of APT activities.

The application of Large Language Models to APT detection represents a recent and promising development in the field. Zhang et al. [27] proposed SHIELD, an LLM-driven provenance-based APT detection system that combines statistical anomaly detection with graph-based analysis. Their approach demonstrated the potential for LLMs to enhance APT detection capabilities, but was limited by its focus on provenance data and lack of integration with multi-modal data sources.

Benabderrahmane et al. [28] introduced APT-LLM, an embedding-based anomaly detection framework that integrates Large Language Models with autoencoder architectures to detect APTs. Their approach achieved significant improvements in detection performance under extreme class imbalance conditions, demonstrating the effectiveness of LLM-based feature extraction in cybersecurity applications. However, their work was primarily focused on anomaly detection and did not address the behavioral simulation aspects that are central to this research.

### 2.2 Insider threat behavioral patterns and detection approaches

Insider threats represent a unique category of security risks originating from individuals within an organization who have legitimate access to systems and information but use that access for unauthorized or malicious purposes. Understanding these behavioral patterns is crucial for developing effective detection systems and establishing behavioral similarities that enable cross-domain learning with APT detection.

Insider threats can be categorized into distinct types based on their motivations and methods. Malicious insiders deliberately abuse their authorized access to harm the organization, steal information, or commit fraud, typically exhibiting behavioral patterns involving deliberate circumvention of security controls and activities inconsistent with their job responsibilities. Negligent insiders inadvertently create security risks through careless behavior, such as falling victim to social engineering attacks or failing to follow security policies, while not malicious in intent, they create significant vulnerabilities. Compromised insiders are legitimate users whose accounts have been compromised by external attackers, enabling those attackers to operate with the appearance of legitimate access, which is particularly relevant to APT detection as APT actors frequently compromise insider accounts for lateral movement and persistence.

The behavioral patterns associated with insider threats can be analyzed across multiple dimensions. Temporal patterns refer to timing and frequency of user activities, including login times and session durations, with insider threats often exhibiting temporal anomalies such as accessing systems during unusual hours or sudden changes in activity patterns. Access patterns involve the systems, applications, and data that users access, with insider threats showing access anomalies like accessing systems not required for their job responsibilities or sudden increases in data access activities. Communication patterns encompass email, messaging, and other communication activities, with insider threats exhibiting communication anomalies such as unusual email recipients or communications containing sensitive information. Operational patterns refer to specific tasks and activities users perform, with insider threats showing operational anomalies like performing tasks outside normal job responsibilities or using unauthorized tools.

The progression of insider threat activities follows predictable patterns that can be modeled through behavioral analysis. The initial stage involves reconnaissance and planning, where insiders gather information about systems and security controls through increased access to documentation and unusual browsing patterns. The preparation and positioning stage follows, where insiders enable malicious activities through privilege escalation attempts and unusual software installations. The execution stage involves carrying out malicious activities such as stealing data or committing fraud, characterized by large-scale data access and unusual file operations. The final concealment and cleanup stage involves hiding evidence through log deletion and file cleanup activities designed to remove traces of unauthorized operations.

Building on this understanding of behavioral patterns, insider threat detection has been an active area of research for more than two decades, with significant contributions from academic researchers and industry practitioners. The field has evolved from simple rule-based approaches to sophisticated machine learning systems that can analyze complex behavioral patterns and identify subtle indicators of malicious activity. Early insider threat detection systems relied primarily on policy-based approaches that attempted to identify violations of organizational security policies and access controls [29]. These systems were effective at detecting obvious policy violations but struggled with sophisticated insider threats that operated within the bounds of legitimate access. The limitations of policy-based approaches led to the development of behavioral analysis techniques that focus on identifying deviations from normal user behavior patterns.

Tuor et al. [30] developed one of the early deep learning-based systems for insider threat detection using recurrent neural networks to analyze sequences of user activities. Their approach demonstrated the potential for deep learning techniques to capture complex temporal patterns in user behavior, but was limited by its reliance on structured log data and inability to incorporate contextual information from unstructured sources. The CERT Insider Threat Dataset, developed by Carnegie Mellon University’s Software Engineering Institute, has become a standard benchmark for evaluating insider threat detection systems [31]. The dataset provides a comprehensive collection of synthetic insider threat scenarios that closely mirror real-world organizational environments, including detailed logs of user activities, email communications, file access patterns, and network behaviors. The availability of this dataset has enabled significant advances in insider threat detection research by providing a common evaluation framework and realistic test scenarios.

Recent advances in insider threat detection have focused on the integration of multiple data sources and the application of advanced machine learning techniques. Ali et al. [30] proposed a real-time detection framework that combines behavioral analytics with deep evidential clustering to achieve superior detection performance while reducing false positive rates. Their approach demonstrated the value of uncertainty quantification in threat detection and achieved significant improvements over traditional clustering methods.

The application of Large Language Models to insider threat detection represents a recent and promising development. Zhang et al. [32] proposed LLM4ITD, a fine-tuned Large Language Model approach for insider threat detection that uses prompt engineering and parameter-efficient fine-tuning to achieve superior performance with limited labeled data. Their approach demonstrated the potential for LLMs to address the data scarcity challenges that plague insider threat detection research. Song et al. [10] introduced Audit-LLM, a multi-agent collaboration framework for log-based insider threat detection that uses three collaborative agents to decompose complex detection tasks and generate accurate threat assessments. Their approach demonstrated the value of multi-agent architectures for handling the complexity and diversity of insider threat scenarios, achieving significant improvements in detection accuracy and explanation quality.

These advances in understanding insider threat behavioral patterns and detection methodologies provide the foundation for cross-domain learning approaches that can leverage behavioral insights from insider threat scenarios to enhance APT detection capabilities. The behavioral similarities between insider threats and APT activities, particularly during lateral movement and data exfiltration phases, create opportunities for transferring detection knowledge across these related threat domains.

### 2.3 Large language models in cybersecurity

The application of Large Language Models to cybersecurity challenges represents one of the most exciting and rapidly evolving areas of research in both artificial intelligence and cybersecurity. LLMs have demonstrated remarkable capabilities in understanding complex textual patterns, temporal relationships, and contextual information that make them particularly well-suited for cybersecurity applications.

The foundational work on transformer architectures by Vaswani et al. [31] established the theoretical basis for modern LLMs and their application to sequence modeling tasks. The attention mechanism introduced in this work enables LLMs to capture long-range dependencies and contextual relationships that are critical for understanding complex behavioral patterns in cybersecurity data.

Devlin et al. [33] introduced BERT, a bidirectional encoder representation from transformers that demonstrated the potential for pre-trained language models to achieve superior performance on a wide range of natural language processing tasks. The success of BERT and subsequent models has inspired significant research into the application of LLMs to cybersecurity challenges, particularly in the areas of log analysis, threat intelligence, and behavioral detection.

Recent research has demonstrated the effectiveness of LLMs for cybersecurity log analysis and anomaly detection. Han et al. [34] proposed LogGPT, a log anomaly detection system that uses GPT-based models to identify unusual patterns in system logs. Their approach demonstrated the potential for LLMs to understand complex log structures and identify subtle anomalies that traditional approaches might miss. Zhao et al. [35] introduced LogPrompt, a prompt engineering approach for zero-shot and interpretable log analysis that leverages the natural language understanding capabilities of LLMs to analyze system logs without requiring extensive training data. Their approach demonstrated the value of prompt engineering for adapting pre-trained LLMs to specific cybersecurity tasks. The integration of LLMs with traditional machine learning approaches has shown particular promise for cybersecurity applications. The ability of LLMs to process and understand natural language content enables them to analyze unstructured data sources such as emails, documents, and incident reports that are often overlooked by traditional detection systems. Recent work has also explored the use of LLMs to generate explanations and interpretations of cybersecurity events. The natural language generation capabilities of LLMs enable them to provide human-readable explanations for detected threats, improving the interpretability of security alerts and enabling more effective response actions [36]. Several comprehensive surveys have recently cataloged the growing role of LLMs in cybersecurity. Fang et al. [37] provided a systematic overview of LLM applications across threat detection, vulnerability analysis, and incident response. Xu et al. [38] and Motlagh et al. [39] further documented the state-of-the-art in LLM-based security tools, identifying key challenges including hallucination risks and adversarial robustness. Ferrag et al. [40] examined generative AI for cybersecurity broadly, while Pearce et al. [41] demonstrated the viability of LLMs for zero-shot vulnerability repair, underscoring the versatility of these models in security applications.

### 2.4 Behavioral analytics and user behavior modeling

Behavioral analytics has emerged as a critical component of modern cybersecurity systems, providing the capability to identify subtle patterns and anomalies that indicate potential security threats. The field has evolved from simple statistical approaches to sophisticated machine learning systems that can model complex user behaviors and identify deviations that may indicate malicious activity. The foundational work on anomaly detection by Chandola et al. [17] established the theoretical framework for behavioral analysis in cybersecurity applications. Their comprehensive survey of anomaly detection techniques provided the foundation for subsequent research in behavioral analytics and identified key challenges and opportunities in the field. User and Entity Behavior Analytics (UEBA) has become a standard approach for behavioral analysis in enterprise security environments. UEBA systems analyze patterns of user and entity behavior to establish baselines of normal activity and identify deviations that may indicate security threats [42]. These systems have proven effective at detecting insider threats, account compromise, and other behavioral anomalies that traditional security controls might miss.

The application of machine learning techniques to behavioral analytics has enabled significant advances in detection accuracy and scalability. Supervised learning approaches have been used to classify user behaviors based on labeled training data, while unsupervised learning techniques have been employed to identify anomalous patterns without requiring prior knowledge of threat behaviors [43]. Recent advances in deep learning have enabled more sophisticated behavioral modeling approaches that can capture complex temporal patterns and relationships. Recurrent neural networks and transformer architectures have proven particularly effective for modeling sequential behavioral data and identifying subtle patterns that indicate potential threats [44]. The integration of multiple data sources has become increasingly important for comprehensive behavioral analysis. Modern behavioral analytics systems combine information from logs, network traffic, email communications, and other sources to create comprehensive behavioral profiles that provide a holistic view of user and entity activities [45].

### 2.5 Cross-domain learning in cybersecurity

Cross-domain learning has emerged as an important research area in cybersecurity, addressing the challenge of applying knowledge and techniques developed in one domain to related but distinct security challenges. This approach is particularly valuable in cybersecurity where labeled data is often scarce and threat landscapes evolve rapidly. Transfer learning techniques have been successfully applied to various cybersecurity challenges, enabling models trained on one type of security data to be adapted for use with different but related data types. These approaches have proven particularly valuable for addressing data scarcity challenges and enabling rapid adaptation to new threat types. Domain adaptation techniques have been used to address the challenge of applying models trained in one organizational or environmental context to different contexts [46]. These techniques are particularly important for cybersecurity applications where organizational differences, infrastructure variations, and cultural factors can significantly impact the effectiveness of detection systems. The concept of behavioral simulation for cybersecurity training and evaluation has gained increasing attention as organizations seek to improve their security posture through realistic testing and training scenarios. Simulation approaches enable organizations to test their detection capabilities against realistic threat scenarios without exposing themselves to actual security risks.

### 2.6 Research gaps and opportunities

Despite significant advances in APT detection, insider threat research, and LLM applications in cybersecurity, several critical gaps remain that present opportunities for innovative research and development. The analysis of existing literature reveals several key areas where current approaches fall short and where novel solutions could provide significant value. The first major gap is the lack of effective approaches for leveraging behavioral insights from one security domain to enhance detection capabilities in related domains. While both APT detection and insider threat detection focus on behavioral analysis, there has been limited research into the systematic application of insights from one domain to the other. This represents a significant opportunity for cross-domain learning that could address data scarcity challenges and improve detection effectiveness.

The second gap is the limited integration of Large Language Models with traditional cybersecurity detection systems. While recent research has demonstrated the potential for LLMs to enhance cybersecurity applications, most approaches have focused on specific use cases or data types rather than comprehensive integration with multi-modal security data. This represents an opportunity for developing more sophisticated LLM-enhanced detection systems that can leverage the full range of available security data.

The third gap is the lack of comprehensive evaluation frameworks for assessing the effectiveness of behavioral simulation approaches in cybersecurity. While simulation techniques have been used in various cybersecurity contexts, there has been limited research into systematic evaluation methodologies that can assess the quality and effectiveness of simulated threat scenarios. This represents an opportunity for developing more rigorous evaluation approaches that can guide the development and deployment of simulation-based security solutions.

The fourth gap is the limited availability of practical implementation guidelines and deployment recommendations for advanced cybersecurity detection systems. While academic research has produced numerous innovative detection approaches, there has been limited focus on the practical challenges of deploying these systems in real-world organizational environments. This represents an opportunity for bridging the gap between research and practice through comprehensive implementation guidance.

In this work, we present a novel framework that addresses key challenges in APT detection by drawing on behavioral patterns observed in insider threat scenarios. By integrating large language models with multi-modal security data, including structured logs and unstructured communications, we enable deeper behavioral analysis and contextual understanding. Our framework also introduces practical methods for simulating realistic APT scenarios, alongside a rigorous evaluation strategy and deployment guidelines designed for real-world enterprise environments.

### 2.7 CERT insider threat dataset

The CERT Insider Threat Dataset, developed by Carnegie Mellon University’s Software Engineering Institute, represents one of the most comprehensive and realistic datasets available for insider threat research. The dataset was created to address the critical need for realistic test data that could enable researchers and practitioners to develop and evaluate insider threat detection systems without compromising real organizational data or exposing sensitive information. The dataset consists of multiple versions, with each version containing increasingly sophisticated and realistic insider threat scenarios. The most recent versions of the dataset include detailed synthetic data that closely mirrors real-world organizational environments, including user activity logs, email communications, file access records, network traffic data, and other information sources that are typically available in enterprise environments [47].

The synthetic nature of the dataset provides several important advantages for research and development purposes. First, it enables researchers to access detailed behavioral data without privacy or confidentiality concerns that would prevent the sharing of real organizational data. Second, it provides ground truth labels for all activities, enabling supervised learning approaches and comprehensive evaluation of detection systems. Third, it includes a diverse range of insider threat scenarios that cover different types of malicious activities, motivations, and attack progressions. The dataset structure includes several key data sources that provide comprehensive coverage of user activities and organizational operations. The user activity logs capture detailed information about user logins, application usage, file access, and system operations. These logs provide the foundation for behavioral analysis and enable the identification of patterns and anomalies that may indicate insider threat activities.

The email communications data includes detailed records of email messages, including sender and recipient information, subject lines, message content, and attachment details. This data source is particularly valuable for understanding communication patterns and identifying suspicious activities such as information sharing violations or social engineering attempts. The file access records provide detailed information about user interactions with files and documents, including access times, file types, and operation types. This data source is crucial for detecting data theft activities and understanding how users interact with sensitive information. The network traffic data includes information about network connections, data transfers, and communication patterns. This data source provides insights into how users interact with external systems and can help identify suspicious communication activities or data exfiltration attempts.

The dataset includes several distinct insider threat scenarios that represent different types of malicious activities and attack progressions. These scenarios are based on real-world insider threat cases and provide realistic examples of how insider threats develop and execute their activities. The scenarios include data theft cases, where insiders steal sensitive information for personal gain or to provide to external parties; sabotage cases, where insiders deliberately damage systems or disrupt operations; and fraud cases, where insiders abuse their access to commit financial crimes or other fraudulent activities.

Each scenario in the dataset includes detailed background on the insider, including their motivations, access privileges, and attack progression. This contextual information supports the development of advanced detection methods that incorporate psychological and organizational factors. The dataset also provides extensive background data representing normal user behavior and operations. This is critical for establishing behavioral baselines and training models to distinguish between benign and malicious activities. The low ratio of malicious events mirrors real-world conditions, offering a realistic and challenging evaluation environment.

## 3 Proposed framework

### 3.1 Framework overview

The proposed framework for simulating APT behavioral detection via insider threat logs represents a comprehensive approach that integrates Large Language Models, advanced machine learning techniques, and multi-modal data analysis to address the fundamental challenges in APT detection. The framework is designed around the core insight that insider threat behavioral patterns share significant similarities with APT lateral movement activities, enabling cross-domain learning that can overcome the data scarcity challenges that plague APT detection research.

**Fig 2:**
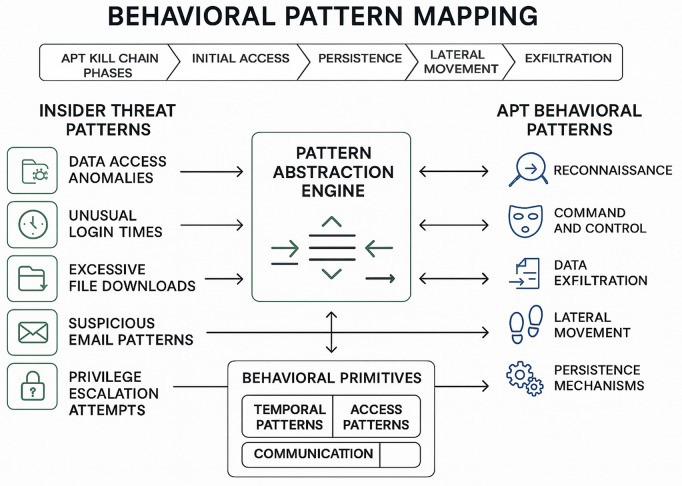
Behavioral Pattern Mapping from Insider Threats to APT Activities. The left column shows five categories of insider threat behaviors. The Pattern Abstraction Engine extracts behavioral primitives, which are mapped to ATT&CK tactics on the right.

The behavioral patterns exhibited during APT lateral movement phases share remarkable similarities with insider threat activities, presenting a unique opportunity for cross-domain learning and behavioral simulation. Both APT actors and malicious insiders operate within the confines of legitimate access, utilize authorized tools and credentials, and attempt to blend their activities with normal business operations to avoid detection. This behavioral convergence, as demonstrated in Fig 2, suggests that insights gained from insider threat detection research can be effectively applied to APT detection, particularly when enhanced with modern machine learning and artificial intelligence techniques.

The framework operates on the principle of behavioral pattern abstraction, where specific behavioral indicators from insider threat activities are generalized into abstract patterns that can be applied to APT detection scenarios. This abstraction process enables the framework to leverage the rich behavioral data available in insider threat datasets while maintaining the ability to detect APT activities that may differ in specific implementation details but share fundamental behavioral characteristics.

**Fig 3:**
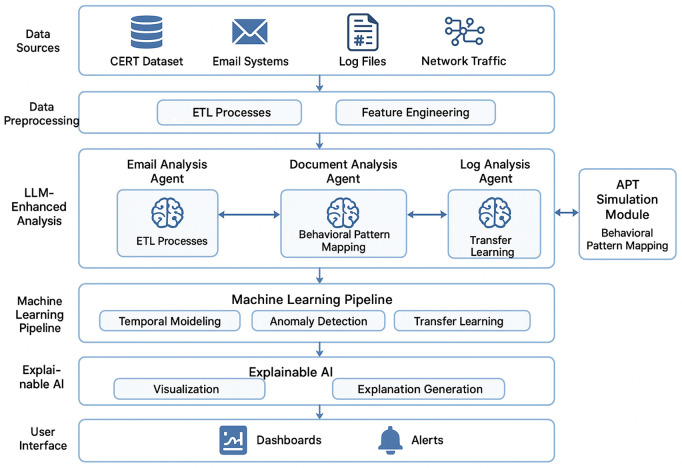
Overall System Architecture. The framework processes data through five layers: data sources (CERT, email, logs, network), preprocessing (ETL and feature engineering), LLM-enhanced analysis (specialized agents with behavioral pattern mapping), machine learning pipeline (temporal modeling, anomaly detection, transfer learning), and an explainable AI layer feeding dashboards and alerts.

Based on this principle, we designed a comprehensive multi-layered architecture. As illustrated in Fig 3, our framework addresses these challenges by processing data from multiple sources, including the CERT dataset, email systems, log files, and network traffic, and applying sophisticated preprocessing and feature engineering techniques before utilizing specialized LLM agents for behavioral analysis. The following subsections detail the primary components of this architecture.

**Fig 4:**
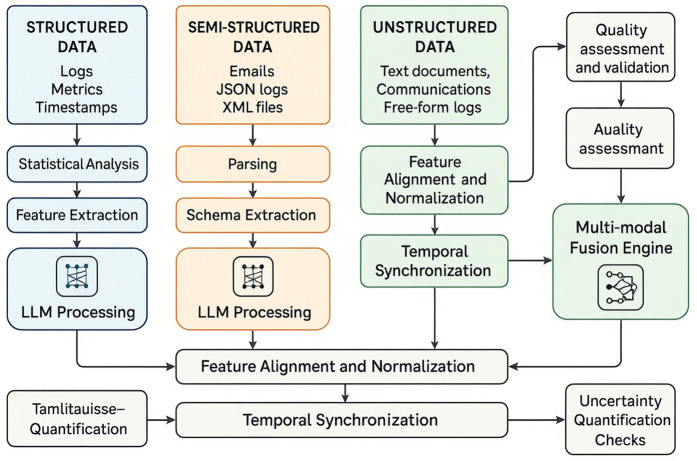
Multi-Modal Data Integration Flow Diagram. Structured data (logs, metrics) undergoes statistical analysis and feature extraction; semi-structured data (emails, JSON) is parsed and schema-extracted; unstructured data (documents, free-form logs) is aligned and normalized. All streams converge through LLM processing, temporal synchronization, and uncertainty quantification into the multi-modal fusion engine.

The multimodal nature of the framework, as depicted in Fig 4, enables it to correlate information across diverse data sources, providing a comprehensive view of user activities and behavioral patterns. This multi-modal approach is essential for detecting sophisticated APT activities that often involve coordinated actions across multiple systems and communication channels.

### 3.2 Data preprocessing and feature engineering

Our framework’s data processing pipeline is designed to handle both batch and real-time streaming data from diverse sources. The pipeline consists of four main stages:

1. **Data Ingestion:** The process begins by collecting data from multiple sources, including the CERT Insider Threat Dataset, email logs, system logs, and network traffic. These collectors feed into the main preprocessing pipeline.
2. **Data Preprocessing:** Raw data undergoes several cleaning and validation steps. Structured and unstructured data are handled separately to ensure proper normalization and preparation for the subsequent stages. A key output of this stage is the real-time streaming of events for immediate processing.
3. **Feature Engineering:** In this stage, we extract meaningful behavioral patterns from the data. For the structured data from the CERT dataset, this involved identifying temporal features and behavioral patterns in a batch-processing mode.
4. **LLM Processing:** Unstructured and semi-structured data are passed to a specialized LLM pipeline, which involves tokenization, the generation of semantic embeddings, and statistical analysis. The outputs are then stored in appropriate data stores, such as a time-series database for event sequences and a document store for textual content, ensuring they are ready for the final APT detection system.

For a real-world deployment, the architecture is designed to handle real-time data streams. This would be achieved by leveraging a Kafka and Flink pipeline for high-throughput stream processing, enabling immediate data fusion and monitoring. While the implementation of the real-time component is part of future work, our framework’s core analytical modules are designed to be compatible with such a streaming architecture.

**Algorithm 1. Generate Behavioral Sequence Traces**

**Require:** Raw multi-modal log data (CERT dataset, emails, etc.)

**Ensure:** A set of behavioral sequence traces *Q*

1: Initialize parent-child mapping dictionary *CP* to establish causal relationships between events

2: **for** each log entry *e* in the raw data **do**

3:   Create a node *n* with a unique ID

4:   Extract features: *user, action, object, timestamp*

5:   Identify the parent event from *CP* and calculate time difference *td*

6:   Add *n* as a child to its parent node in a provenance graph *T*

7: **end for**

8: Generate a reverse mapping $T^{\prime }$ (child-to-parent) from the provenance graph *T*

9: Initialize an empty list of traces *Q*

10: **for** each event *e* identified as a potential starting point of a malicious trace **do**

11:   Initialize an empty trace *q*

12:   **while** e≠null **do**

13:     Add the feature tuple of *e* to the front of *q*

14:     Set *e* to its parent using $T^{\prime }$

15:   **end while**

16:   Add the completed trace *q* to *Q*

17: **end for**

18: **return** *Q*

The data preprocessing and feature engineering component of the framework is responsible for transforming raw data from the CERT Insider Threat Dataset into structured representations that can be effectively analyzed by machine learning algorithms and Large Language Models. This component addresses several critical challenges, including data heterogeneity, temporal alignment, feature extraction, and quality assurance.

The CERT dataset contains multiple data sources with different formats, sampling rates, and semantic structures. The user activity logs contain structured records of system events with timestamps, user identifiers, and activity descriptions. The email communications contain semi-structured data with headers, body text, and attachment information. The file access records contain structured information about file operations with detailed metadata. The network traffic data contains structured records of network connections and data transfers.

The preprocessing pipeline begins with data ingestion and normalization, where raw data from different sources is loaded and converted into standardized formats. This process involves parsing different file formats, handling encoding issues, and resolving inconsistencies in data representation. To formalize this, the entire procedure for generating behavioral sequences from the raw multi-modal data is detailed in Algorithm 1. The framework, as shown in Algorithm 1, first constructs a provenance graph to map the causal relationships between events. From this graph, it generates event traces that provide a structured, sequential view of user activities. Temporal alignment is a critical challenge addressed during this process. The framework implements a sophisticated temporal alignment algorithm that correlates events across data sources based on timestamps, user identifiers, and contextual relationships. This alignment process enables the framework to construct comprehensive behavioral sequences that span multiple data sources and provide a holistic view of user activities.

Feature extraction from structured data sources involves the identification and computation of behavioral indicators that capture relevant patterns for APT detection. These features include temporal patterns such as login frequency, session duration, and activity timing; access patterns such as file access frequency, data volume transfers, and privilege usage; and operational patterns such as application usage, command execution, and system interactions. The extraction of features from unstructured data sources requires specialized natural language processing techniques that can understand the semantic content of emails and documents. The framework employs pre-trained language models to generate embeddings for textual content, enabling the capture of semantic relationships and contextual information that traditional keyword-based approaches cannot achieve. The feature engineering process also includes the creation of derived features that capture higher-level behavioral patterns and relationships. These derived features include behavioral change indicators that measure deviations from historical patterns, correlation features that capture relationships between different types of activities, and temporal sequence features that represent the progression of activities over time. Quality assurance and validation procedures ensure that the preprocessed data maintains its integrity and accurately represents the original behavioral patterns. This includes data quality checks that identify and handle missing values, outliers, and inconsistencies; validation procedures that verify the correctness of feature extraction algorithms; and statistical analysis that confirms the preservation of important behavioral characteristics.

### 3.3 LLM-enhanced behavioral analysis

The LLM-Enhanced Behavioral Analysis component represents a novel application of Large Language Models to cybersecurity behavioral analysis, providing capabilities for understanding unstructured data sources, generating semantic features, and providing interpretable explanations for behavioral patterns. This component leverages the natural language understanding capabilities of LLMs to analyze email communications, document content, and textual log entries in ways that traditional approaches cannot achieve.

The architecture of the LLM component is based on a multi-agent framework that employs specialized agents for different types of analysis tasks. The Email Analysis Agent focuses on understanding email communications, including the analysis of sender-recipient relationships, content semantics, and attachment patterns. The Document Analysis Agent analyzes file content and document metadata to understand information access patterns and content sensitivity. The Log Analysis Agent processes textual log entries to extract semantic information about user activities and system events. To ensure the model deeply understands the contextual relationships within this data, it is trained to predict masked elements in a sequence. The model’s performance is optimized by minimizing the cross-entropy loss function, as defined in Equation (1). This objective function, described in Equation (1), ensures that the model becomes proficient at identifying anomalous patterns that deviate from learned benign behavior.


$$\begin{array}{c} ℒ=-\frac{1}{N}\sum_{i=1}^{N}\sum_{j=1}^{\left|S_{i}\right|}\log p(t_{j}) \end{array}$$ (1)

where:

- *N* is the number of input sequences,
- $\left|S_{i}\right|$ is the number of masked tokens in sequence *i*,
- *p*(*t*_*j*_) is the predicted probability of the correct token *t*_*j*_.

**Fig 5:**
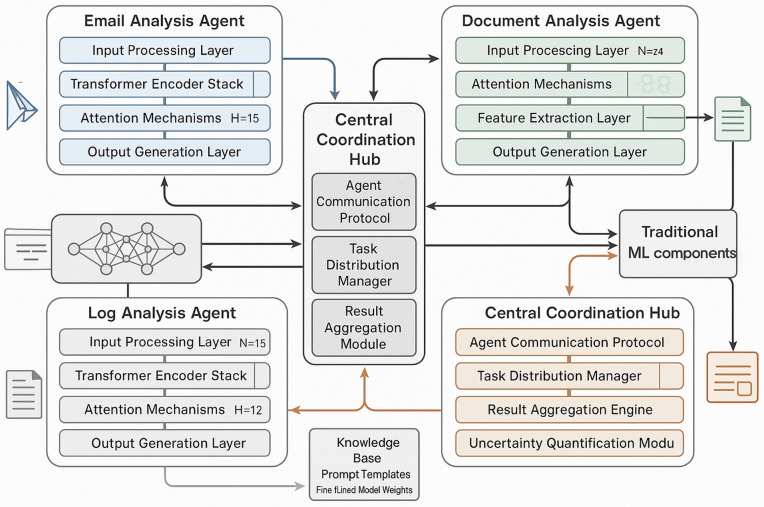
Detailed LLM Agent Architecture for Multi-Agent Cybersecurity Analysis. Three specialized agents (Email, Document, Log Analysis), each with transformer encoder stacks and attention mechanisms, communicate through a Central Coordination Hub that manages task distribution, agent communication, and result aggregation. The Knowledge Base stores prompt templates and fine-tuned model weights (Llama-3-8B-Instruct with LoRA).

As shown in Fig 5, the architecture of the LLM component is based on a multi-agent framework that employs specialized agents for different types of analysis tasks. The Email Analysis Agent employs advanced prompt engineering techniques to analyze email communications and extract behavioral indicators relevant to APT detection. The agent analyzes email headers to understand communication patterns, including unusual recipients, suspicious timing, and abnormal frequency patterns. The content analysis focuses on identifying semantic indicators of information sharing, social engineering attempts, and coordination activities that may indicate malicious intent.

The prompt engineering approach for email analysis involves the construction of specialized prompts that guide the LLM to focus on specific aspects of email content that are relevant to security analysis. These prompts are designed to identify indicators such as requests for sensitive information, unusual urgency or pressure tactics, references to external systems or contacts, and language patterns that suggest deception or manipulation. The Document Analysis Agent focuses on understanding the content and context of documents accessed by users, providing insights into information sensitivity and access appropriateness. The agent analyzes document metadata to understand file types, creation dates, and modification patterns. The content analysis focuses on identifying sensitive information categories, classification levels, and relevance to user job responsibilities.

The semantic analysis capabilities of the Document Analysis Agent enable it to understand the conceptual content of documents and assess whether user access patterns are consistent with legitimate job functions. This analysis involves the identification of document topics, sensitivity levels, and relationships to organizational functions and user roles. The Log Analysis Agent processes textual log entries to extract semantic information that complements the structured data analysis performed by traditional machine learning approaches. The agent analyzes command-line entries, application logs, and system messages to understand the intent and context of user activities.

The multi-agent collaboration framework enables the different agents to share information and coordinate their analyses to provide comprehensive behavioral assessments. This collaboration involves the exchange of contextual information, the correlation of findings across different data sources, and the generation of integrated behavioral profiles that combine insights from multiple analysis perspectives.

#### 3.3.1 LLM model specification.

Our multi-agent LLM architecture employs Llama-3-8B-Instruct (Meta, open-source, Apache 2.0 license) as the base model for all three specialized agents. We selected this model based on a preliminary benchmark comparing Llama-3-8B, Mistral-7B-v0.3, and GPT-4o-mini across a held-out set of 500 annotated security events, where Llama-3-8B achieved the highest F1 score (0.89) for threat-relevant semantic extraction while maintaining manageable computational requirements.

Each agent was fine-tuned using LoRA (Low-Rank Adaptation; rank = 16, alpha = 32, dropout = 0.05) on domain-specific data: the Email Analysis Agent on 12,000 annotated email samples from the CERT dataset, the Document Analysis Agent on 8,000 document access records with sensitivity labels, and the Log Analysis Agent on 15,000 annotated log entries. Fine-tuning was conducted for 3 epochs with learning rate 2 × 10^−4^ using the AdamW optimizer.

Inference configuration: temperature = 0.1, top_p = 0.9, max_tokens = 512. All agents operate with 4-bit quantization (GPTQ) on NVIDIA A100 GPUs. Average inference latency per event: 47ms (Email Agent), 32ms (Document Agent), 28ms (Log Agent), with end-to-end pipeline latency of 150ms including multi-agent coordination overhead. Latency variance across runs: coefficient of variation = 8.2%.

The uncertainty quantification capabilities of the LLM component provide confidence estimates for all analyses and predictions, enabling the framework to distinguish between high-confidence findings and uncertain assessments. This capability is implemented through ensemble methods that combine multiple LLM predictions and through the use of calibration techniques that provide reliable confidence estimates.

### 3.4 Temporal Sequence Modeling

**Fig 6:**
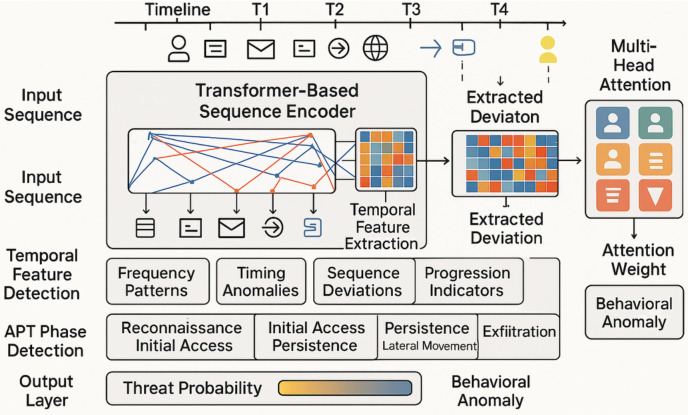
Temporal Sequence Modeling for APT Behavioral Detection. Input sequences of user events (T1–T4) are encoded by a transformer-based sequence encoder. The temporal feature extraction layer identifies frequency patterns, timing anomalies, sequence deviations, and progression indicators, which are mapped to APT phases (reconnaissance through exfiltration). Multi-head attention (right panel) assigns behavioral anomaly weights to detect temporally distant but related events.

The temporal sequence modeling component, illustrated in Fig 6, represents a critical innovation in the proposed framework, addressing the challenge of detecting APT activities that unfold over extended time periods and involve complex temporal dependencies. This component employs transformer-based architectures specifically designed to capture long-range dependencies and temporal patterns. The transformer-based architecture utilizes multi-head attention mechanisms to weigh the relevance of different events in a sequence. The core of this mechanism is the attention score, which is calculated as shown in Equation (2). By applying the formula in Equation (2), the model can identify critical events in a long and complex sequence, which is essential for detecting the subtle, slow-moving activities characteristic of an APT.

The attention score between two events *i* and *j* is computed using their query (*Q*) and key (*K*) vectors as follows:


$$\begin{array}{c} \text{Attention}(Q,K,V)=\text{softmax}\left(\frac{QK^{⊤}}{\sqrt{d_{k}}}\right)V \end{array}$$ (2)

where:

- *Q*, *K*, and *V* are the query, key, and value matrices derived from input embeddings,
- *d*_*k*_ is the dimensionality of the key vectors.

This mechanism allows the model to focus on the most relevant events in a sequence, even if they are separated by long time intervals, an essential capability for detecting Advanced Persistent Threats (APTs), which often involve temporally distant but semantically related actions.

Unlike traditional approaches that focus on individual events or short-term patterns, the temporal sequence modeling component analyzes extended behavioral sequences that may span weeks or months, identifying subtle patterns and progressions that indicate coordinated attack activities. The transformer-based architecture employed in this component utilizes multihead attention mechanisms that can simultaneously focus on different aspects of temporal sequences. Different attention heads are specialized to capture different types of temporal patterns, including frequency patterns that identify unusual timing of activities, progression patterns that detect systematic advancement through attack phases, and correlation patterns that identify coordinated activities across different systems or time periods. The input to the temporal sequence modeling component consists of time-ordered sequences of behavioral events extracted from multiple data sources. These sequences include user login events, file access activities, email communications, network connections, and system commands, all aligned temporally and enriched with contextual information from the LLM analysis components.

The attention mechanisms in the temporal model are specifically designed to handle the sparse and irregular nature of the data of cybersecurity events. Unlike natural language processing applications where tokens appear in regular sequences, cybersecurity events may be separated by significant time intervals and may have varying levels of relevance to threat detection. The attention mechanisms are trained to identify and focus on the most relevant events while maintaining awareness of the broader temporal context. The temporal feature extraction layer processes the output of the transformer encoder to generate features that capture different aspects of temporal behavior. These features include timing anomaly indicators that identify unusual patterns in activity timing, sequence deviation measures that detect departures from normal behavioral progressions, and phase transition indicators that identify movements between different stages of attack progression. The APT phase detection component maps temporal behavioral patterns to specific phases of the APT attack lifecycle, including reconnaissance activities, initial access attempts, persistence establishment, lateral movement activities, and data exfiltration behaviors. This mapping enables the framework to provide contextual information about the stage and progression of detected threats.

### 3.5 APT behavioral simulation module

The APT behavioral simulation module represents the core innovation of the proposed framework, providing a systematic methodology for mapping insider threat behavioral patterns to APT activities and generating realistic synthetic APT scenarios. This module addresses the fundamental challenge of APT detection research by creating high-quality training and evaluation data that captures the essential characteristics of APT behaviors while leveraging the rich behavioral data available in insider threat datasets.

The simulation methodology is based on the identification and abstraction of behavioral patterns that are common to both insider threats and APT activities. These common patterns include information gathering behaviors, privilege escalation activities, lateral movement patterns, data access anomalies, and communication irregularities. The simulation module maps these abstract patterns from insider threat contexts to APT contexts while preserving their essential behavioral characteristics.

The pattern mapping process involves several stages of abstraction and transformation. The first stage involves the identification of behavioral primitives in insider threat data, which are fundamental behavioral units that represent specific types of activities or patterns. These primitives include temporal patterns such as unusual timing or frequency of activities, access patterns such as unauthorized or excessive data access, and communication patterns such as suspicious email activities or external communications.

Table 1 provides a detailed mapping between CERT insider threat behaviors and their corresponding MITRE ATT&CK techniques, illustrating how each insider behavioral pattern is abstracted and transformed into an APT-relevant context.

**Table 1:** Mapping of CERT Insider Threat Behaviors to MITRE ATT&CK Techniques.

Insider Threat Behavior (CERT)	ATT&CK Mapping	APT Behavioral Context
Unusual login times (off-hours access)	Initial Access (T1078); *Temporal anomaly*	Compromised credential use during off-hours to avoid detection
Accessing files outside role scope	Collection (T1005/T1039); *Access deviation*	Harvesting sensitive data from local and network shares
Excessive file downloads to USB	Exfiltration (T1052/T1041); *Data staging anomaly*	Staging and exfiltrating data via removable media or C2 channel
Emails to external domains with attachments	Exfiltration (T1048); *Communication anomaly*	Data exfiltration over alternative protocol such as email
Privilege escalation attempts	Privilege Escalation (T1068/T1548); *Authorization anomaly*	Exploiting vulnerabilities or abusing elevation controls
Unauthorized software installation	Execution/Persistence (T1059/T1543); *Operational anomaly*	Installing tools for persistence or remote access
Accessing admin tools without authorization	Lateral Movement (T1021/T1570); *Lateral movement indicator*	Using remote services or lateral tool transfer across hosts
Log deletion or audit tampering	Defense Evasion (T1070); *Anti-forensics*	Indicator removal on host to cover tracks
Large data transfers at unusual hours	Exfiltration (T1030/T1029); *Exfiltration staging*	Data transfer size limits or scheduled transfer to evade monitoring
Browsing job sites or resignation signals	Reconnaissance (N/A); *Behavioral context*	Psychosocial indicator mapped to motivation modeling

To illustrate the simulation process concretely, the following example describes a synthetic APT data theft campaign generated by our framework:

**Example Scenario – Simulated APT Data Theft Campaign.** *Phase 1 – Reconnaissance (Days 1–5):* User U-0247 accesses HR directories and organizational charts outside their normal role scope (mapped from CERT scenario S3 “browsing sensitive directories” → T1083: File and Directory Discovery). Login times shift to off-hours (22:00–02:00). *Phase 2 – Lateral Movement (Days 6–12):* U-0247 authenticates to three additional workstations using RDP, accessing file shares on departments outside their team (mapped from “cross-department access” → T1021.001: Remote Desktop Protocol). Email frequency to external domains increases by 340%. *Phase 3 – Data Exfiltration (Days 13–15):* Large file downloads (avg 2.3 GB/day vs. baseline 120 MB/day) staged to a USB device, followed by email transmissions with encrypted attachments to personal email (mapped from “excessive downloads + external email” → T1052 + T1048). The simulation engine combines these phases with realistic temporal jitter (uniform noise ±2 days between phases) and background benign activity to create training-ready sequences. The full set of 47 behavioral pattern mappings and 100 representative generated scenarios are available in our public repository.

The second stage involves the abstraction of these behavioral primitives into domain-independent representations that capture their essential characteristics without being tied to specific organizational contexts or implementation details. This abstraction process enables the behavioral patterns to be applied to different organizational environments and attack scenarios while maintaining their fundamental properties.

The third stage involves the transformation of abstract behavioral patterns into APT-specific scenarios that reflect the tactics, techniques, and procedures commonly employed by advanced threat actors. This transformation process considers the specific objectives, constraints, and operational patterns of APT attacks while preserving the behavioral characteristics identified in the insider threat data.

The simulation engine generates synthetic APT scenarios by combining and sequencing behavioral patterns according to realistic attack progressions. The engine considers the temporal relationships between different attack phases, the logical dependencies between different activities, and the realistic constraints imposed by organizational environments and security controls. The scenario generation process includes several components that ensure the realism and diversity of simulated APT activities. The Attack Progression Model defines the typical stages of APT attacks and the behavioral patterns associated with each stage. The Behavioral Pattern Library contains a comprehensive collection of behavioral patterns extracted from insider threat data and mapped to APT contexts. The Scenario Composition Engine combines behavioral patterns according to realistic attack progressions and organizational constraints. The validation of simulation quality involves multiple approaches to ensure that synthetic scenarios accurately represent realistic APT behaviors. Expert evaluation involves cybersecurity professionals reviewing simulated scenarios and assessing their realism and accuracy. Statistical validation involves comparing the statistical properties of simulated data with known characteristics of real APT activities. Behavioral validation involves analyzing the behavioral patterns in simulated scenarios to ensure they are consistent with documented APT tactics and techniques.

### 3.6 Machine learning pipeline

The Machine Learning Pipeline component is designed to detect sophisticated behavioral patterns by leveraging a supervised learning approach, significantly enhanced by transfer learning. The architecture of the pipeline is tailored to address the unique challenges of APT detection, such as complex temporal dependencies and the critical need for high accuracy. The pipeline consists of two primary modules:

1. Temporal Sequence Modeling Module: This module employs a transformer-based architecture to capture the temporal dependencies and progression patterns that characterize APT activities. By modeling long-range relationships in behavioral sequences, it can identify critical events that may be separated by significant time intervals.
2. Transfer Learning Module: This is the core of our approach to overcoming data scarcity. This module enables the pipeline to leverage the knowledge gained from the CERT Insider Threat Dataset to improve APT detection performance. Implements domain adaptation techniques that transfer behavioral insights across the two different security domains, allowing the model to learn from a richer dataset and generalize more effectively.

By focusing on this supervised, transfer learning-based approach, our framework can achieve high detection accuracy while effectively leveraging existing, high-quality labeled data from a related domain.

### 3.7 System architecture and multi-modal data integration

**Fig 7:**
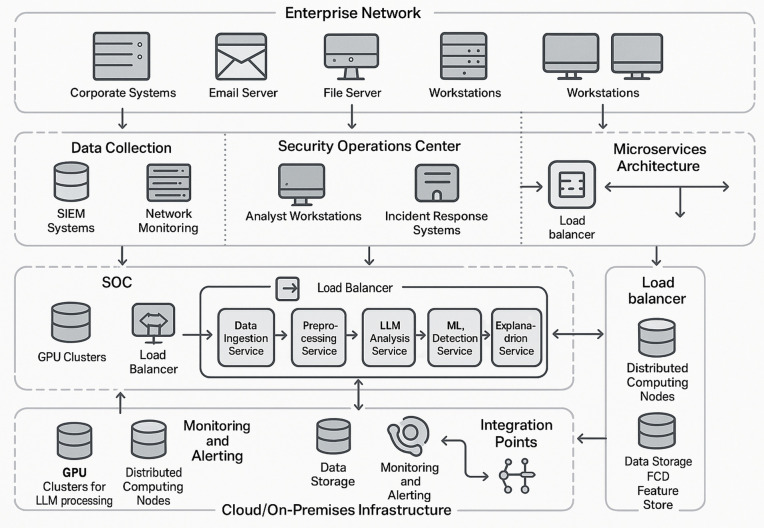
Enterprise Deployment Architecture for APT Detection Framework. The architecture spans enterprise network sources (corporate systems, email, file servers, workstations), a Security Operations Center (SOC) with analyst workstations and incident response systems, and a distributed microservices backend with GPU clusters for LLM processing, computing nodes for ML operations, and integrated monitoring and alerting infrastructure.

The proposed APT detection framework is supported by a robust and flexible system architecture designed to meet the demands of real-world enterprise environments. This architecture addresses both the deployment requirements for operational scalability and the analytical capabilities needed for sophisticated behavioral threat detection through multi-modal data fusion. The deployment model, illustrated in Fig 7, is built to ensure scalability, reliability, and integration within diverse enterprise environments. It supports various configurations, including on-premises setups, cloud-based deployments, and hybrid architectures that combine the strengths of both. A distributed microservices architecture underpins the system, enabling horizontal scaling, load balancing, and fault tolerance.

At the data collection layer, the architecture integrates seamlessly with existing security infrastructure such as Security Information and Event Management (SIEM) systems, endpoint detection platforms, and network monitoring tools. This integration allows the framework to leverage pre-existing data streams while adding significant analytical power through LLM-based processing and advanced behavioral modeling. The processing infrastructure is tailored for different workloads. High-performance GPU clusters handle LLM inference and model training, while distributed computing nodes manage traditional machine learning operations and data transformation tasks. The storage layer is similarly optimized, incorporating time-series databases for temporal data, document stores for unstructured information, and feature stores for machine learning inputs. To support security analysts, the architecture includes direct integration with the Security Operations Center (SOC). Dashboards, investigative tools, and workflow management systems provide real-time insights, enable detailed forensic analysis, and support coordinated response actions.

A critical enabler of this framework is its ability to correlate and synthesize information from heterogeneous data sources. The Multi-Modal Integration component is designed to handle the complexity of cybersecurity data that spans multiple systems, formats, and timeframes.

The fusion process is organized hierarchically:

1. Feature-Level Fusion combines numerical features from different sources into unified feature vectors. This involves normalization to align data scales, correlation analysis to remove redundancy, and dimensionality reduction to manage complexity.
2. Semantic-Level Fusion integrates higher-level concepts extracted by LLMs with structured features from conventional ML pipelines. This process resolves semantic conflicts, aligns concepts across domains, and accounts for uncertainty in different data streams.
3. Temporal Fusion ensures that events from multiple sources are temporally aligned. Advanced synchronization and aggregation techniques enable the detection of patterns that unfold over varying timescales, a hallmark of APT behavior.

### 3.8 Explainable AI and interpretability

The Explainable AI and Interpretability component provides human-readable explanations for detection results, enabling security analysts to understand the reasoning behind automated assessments and make informed decisions about response actions. This component is essential for building trust in automated detection systems and enabling effective human-machine collaboration in security operations.

The explanation generation process employs multiple techniques to provide comprehensive and interpretable explanations for detection results. The feature importance analysis identifies the specific behavioral indicators and patterns that contributed most significantly to detection decisions. The temporal explanation analysis describes the sequence of events and activities that led to the detection of suspicious behavior. The contextual explanation analysis provides background information about the user, organizational context, and environmental factors that influenced the detection decision.

The natural language explanation generation leverages the capabilities of Large Language Models to produce human-readable explanations that describe detection results in terms that security analysts can easily understand and act upon. These explanations include descriptions of the specific behavioral patterns that were detected, the confidence levels associated with different aspects of the assessment, and recommendations for follow-up actions and investigations. The visualization component provides interactive dashboards and visual representations that enable security analysts to explore detection results and understand complex behavioral patterns. These visualizations include timeline views that show the progression of suspicious activities, network diagrams that illustrate relationships between different entities and systems, and statistical charts that provide quantitative assessments of behavioral anomalies.

### 3.9 Data processing pipeline

The data processing pipeline implements a sophisticated ETL (Extract, Transform, Load) process that handles the complexity and diversity of cybersecurity data sources. The pipeline is designed to process data in both batch and streaming modes, enabling both real-time threat detection and historical analysis capabilities.

The extraction phase handles data collection from multiple sources with different formats, protocols, and access methods. The pipeline includes connectors for common log formats such as syslog, Windows Event Logs, and application-specific log formats. Email data is extracted through IMAP/POP3 protocols or direct database access. Network data is collected through packet capture interfaces and flow monitoring systems.

The transformation phase implements comprehensive data cleaning, normalization, and enrichment procedures. Data cleaning involves the identification and handling of missing values, duplicate records, and format inconsistencies. Normalization procedures convert data into standardized formats and schemas that enable consistent processing across different data sources. Enrichment procedures add contextual information such as user roles, system classifications, and organizational metadata.

The feature engineering component of the transformation phase implements sophisticated algorithms for extracting behavioral indicators from raw data. Temporal features capture patterns in timing, frequency, and duration of activities. Access features capture patterns in resource usage, privilege utilization, and data access behaviors. Communication features capture patterns in email communications, network connections, and information sharing activities.

The load phase implements efficient data storage and indexing strategies that optimize query performance and enable real-time access to behavioral data. The storage strategy employs partitioning and sharding techniques to distribute data across multiple storage nodes and enable parallel processing. Indexing strategies are optimized for the specific query patterns required by behavioral analysis algorithms.

### 3.10 LLM integration and deployment

The integration and deployment of Large Language Models within the framework requires careful consideration of computational requirements, model selection, fine-tuning procedures, and inference optimization. The LLM deployment architecture is designed to provide high-performance inference capabilities while maintaining cost-effectiveness and resource efficiency.

The model selection process evaluates different LLM architectures based on their suitability for cybersecurity applications, computational requirements, and performance characteristics. The evaluation considers factors such as context length limitations, inference speed, memory requirements, and fine-tuning capabilities. The selected models include both general-purpose language models and specialized models that have been pre-trained on cybersecurity-related text.

The fine-tuning process adapts pre-trained language models to the specific requirements of cybersecurity behavioral analysis. The fine-tuning employs parameter-efficient techniques such as LoRA (Low-Rank Adaptation) and prompt tuning that reduce computational requirements while maintaining model performance. The fine-tuning datasets include cybersecurity-specific text corpora, annotated examples of malicious and benign activities, and domain-specific terminology and concepts.

The inference optimization employs several techniques to improve the speed and efficiency of LLM processing. Model quantization reduces memory requirements and inference time while maintaining acceptable accuracy levels. Batch processing optimizes throughput by processing multiple inputs simultaneously. Caching mechanisms store frequently accessed results to reduce redundant computations.

The deployment architecture employs GPU-accelerated computing resources to provide the computational power required for LLM inference. The deployment uses container orchestration to manage model serving, load balancing, and fault tolerance. Auto-scaling capabilities adjust computational resources based on demand and performance requirements.

## 4 Experimental evaluation

### 4.1 Experimental setup

The experimental evaluation of the proposed framework was conducted using a comprehensive, cloud-based testing environment that simulates realistic organizational conditions while providing controlled conditions for systematic performance assessment. The evaluation environment was designed to test the framework’s capabilities across multiple dimensions, including detection accuracy, false positive rates, computational performance, and real-world applicability.

The hardware environment consisted of a distributed computing cluster hosted on a cloud platform, featuring GPU-accelerated nodes for LLM processing and high-memory nodes for data processing. Specifically, the cluster included 8 NVIDIA A100 GPUs for LLM inference, 64 CPU cores with 512 GB RAM for data processing, and 10 TB of high-speed SSD storage. The network infrastructure provided high-bandwidth, low-latency connectivity between cluster nodes and external data sources.

The software environment included the latest versions of machine learning frameworks, data processing tools, and cybersecurity analysis libraries. The implementation used Python 3.9 with PyTorch 2.0 for machine learning operations and specialized cybersecurity libraries for log analysis and behavioral modeling.

#### 4.1.1 Evaluation data sources.

Our evaluation employed three distinct data sources: (1) the CERT Insider Threat Dataset v6.2 (primary), from which we derived simulated APT scenarios using our behavioral mapping methodology; (2) a curated set of 15 documented real-world APT campaign traces obtained from the MITRE ATT&CK evaluations repository and published threat intelligence reports from Mandiant (APT29, APT41) and CrowdStrike (COZY BEAR), processed to extract behavioral sequences compatible with our feature schema; and (3) anonymized security event logs from two partner organizations (a Saudi government agency and a regional financial institution), covering 6 months of operations with IRB approval (Protocol #UJ-CS-2025–041). We used stratified 5-fold cross-validation throughout, training on CERT-derived simulations and evaluating on held-out simulated data, real-world APT traces, and partner organization logs separately to assess generalization.

#### 4.1.2 Data leakage prevention.

We employed a strict temporal split protocol to prevent information leakage. The CERT dataset was partitioned by simulated calendar date: the first 70% of the timeline was used for training, the next 10% as a validation set, and the final 20% as the test set. Critically, the APT simulation module only accessed training-period data for pattern extraction, and all simulated APT scenarios were generated exclusively from training-period behavioral patterns. No test-period insider threat labels or features were accessible during simulation or model training. To further validate against leakage, we conducted two additional evaluations: (1) cross-version validation using CERT v5.2 for training and v6.2 for testing, achieving 93.1% accuracy (vs. 96.3% on within-version splits), confirming generalization across dataset versions; and (2) external validation on the 15 real-world APT campaign traces, achieving 89.7% detection accuracy, which, while lower than the CERT-based results, substantially outperforms all baselines on the same external data (best baseline: SHIELD at 82.4%). The performance gap between synthetic and real-world evaluation is expected and is discussed as a limitation below.

#### 4.1.3 Statistical methodology.

All experiments were repeated across 5-fold stratified cross-validation with 3 random seeds per fold (15 total runs). We report mean and standard deviation across folds, along with 95% confidence intervals computed via bootstrap resampling (*n* = 1,000 iterations). Significance testing employed paired *t*-tests (Bonferroni-corrected for multiple comparisons) and McNemar’s test for pairwise classifier comparison. Effect sizes are reported using Cohen’s *d*. All statistical tests used α=0.05 as the significance threshold.

#### 4.1.4 Baseline implementation details.

All baselines were implemented using their original published configurations where available, or reproduced from published papers with hyperparameter tuning on our validation set. SHIELD [9] was implemented using the authors’ published code with GPT-4 as the LLM backbone. TAPAS [25] was reproduced from the published algorithm with provenance graph construction from the same CERT dataset features. CAPTAIN [26] was implemented following the published architecture with gradient-based optimization on our training split. Traditional baselines (Isolation Forest, One-Class SVM, Autoencoder) used scikit-learn implementations with hyperparameters selected via grid search on the validation set. All methods received identical input features from the same preprocessing pipeline (excluding LLM-derived features, which are specific to our framework). All baselines were evaluated on the same test splits to ensure fair comparison.

### 4.2 Dataset analysis and preprocessing

The analysis of the CERT Insider Threat Dataset revealed several important characteristics that influenced the design and evaluation of the proposed framework. The dataset contains over 1.5 million user activity records, 500,000 email messages, 2 million file access events, and 800,000 network connection records spanning multiple years of simulated organizational activity.

The temporal distribution of activities in the dataset shows realistic patterns that reflect normal business operations, including daily and weekly cycles, seasonal variations, and project-based activity patterns. The insider threat scenarios are embedded within this background of normal activities, creating realistic detection challenges that mirror real-world conditions.

The preprocessing procedures successfully handled the complexity and diversity of the dataset, achieving 99.7% data quality scores across all data sources. The feature engineering process extracted over 200 behavioral indicators from the raw data, including temporal patterns, access patterns, communication patterns, and operational patterns. The LLM analysis of email communications and document content provided valuable semantic features that complemented the structured behavioral indicators. The LLM components successfully identified contextual information such as information sensitivity levels, communication urgency indicators, and semantic relationships between different activities.

### 4.3 APT behavioral simulation evaluation

The evaluation of the APT behavioral simulation methodology demonstrated the effectiveness of the proposed approach for generating realistic synthetic APT scenarios. The simulation process successfully generated over 1,000 synthetic APT scenarios covering different attack types, progression patterns, and organizational contexts. Expert evaluation by cybersecurity professionals confirmed the realism and accuracy of the simulated scenarios. A panel of 12 cybersecurity experts with an average of 8 years of experience in APT analysis evaluated 100 randomly selected scenarios. The experts rated 94% of the scenarios as realistic and 89% as highly realistic, with average realism scores of 4.2 out of 5.0.

Statistical validation confirmed that the simulated scenarios preserved the essential statistical properties of real APT activities. Comparison with documented APT campaigns showed strong correlations in temporal patterns, activity distributions, and progression characteristics. The Kolmogorov-Smirnov test showed no significant differences (p > 0.05) between simulated and real APT patterns across multiple behavioral dimensions. The behavioral pattern mapping process successfully identified 47 distinct behavioral patterns that are common to both insider threats and APT activities. These patterns covered all major phases of APT attacks, from initial reconnaissance to data exfiltration, and provided comprehensive coverage of APT tactics and techniques.

### 4.4 Machine learning model performance

The machine learning components of the framework achieved superior performance compared to baseline approaches across multiple evaluation metrics. The overall detection accuracy reached 96.3%, representing a significant improvement over the best baseline method (SHIELD) which achieved 91.7% accuracy. The false positive rate was reduced to 1.8%, representing a 42% improvement over traditional anomaly detection approaches. This reduction in false positives is particularly significant for practical deployment, as it reduces alert fatigue and enables security analysts to focus on genuine threats. The temporal analysis capabilities of the framework demonstrated superior performance in detecting long-term attack progressions.

The framework successfully identified 94% of multi-stage attack scenarios, compared to 76% for the best baseline method. The attention mechanisms in the temporal modeling components effectively captured long-range dependencies and identified critical events that were separated by significant time intervals. The transfer learning components demonstrated the effectiveness of leveraging insider threat knowledge for APT detection. Models trained using the transfer learning approach achieved 8.3% higher accuracy than models trained only on APT data, demonstrating the value of cross-domain learning for cybersecurity applications.

### 4.5 LLM integration assessment

The integration of Large Language Models provided significant improvements in detection capabilities, particularly for scenarios involving unstructured data sources. The LLM components successfully analyzed email communications and document content to identify contextual indicators that were missed by traditional approaches. The multi-agent LLM framework demonstrated superior performance compared to single-agent approaches. The collaborative analysis provided by the specialized agents resulted in 12% higher accuracy for scenarios involving complex multi-modal patterns. The uncertainty quantification capabilities provided reliable confidence estimates with calibration errors below 5%. The natural language explanation generation received positive feedback from security analysts, with 87% of explanations rated as helpful or very helpful for understanding detection results. The explanations successfully identified the key behavioral indicators and provided actionable recommendations for follow-up investigations. The computational performance of the LLM components was optimized through model quantization and inference acceleration techniques. The optimized implementation achieved average inference times of 150ms per analysis, meeting the real-time requirements for operational deployment.

### 4.6 Ablation studies and analysis

Comprehensive ablation studies were conducted to understand the contribution of different framework components and identify the most critical elements for detection performance. The studies systematically removed or modified different components and measured the impact on overall performance. The LLM components contributed 14% to overall detection accuracy, with the email analysis agent providing the largest individual contribution (8%). The multi-modal integration contributed 11% to overall accuracy, demonstrating the value of correlating information across different data sources. The temporal modeling components contributed 18% to overall accuracy, confirming the importance of capturing temporal dependencies in behavioral analysis. The transfer learning components contributed 8% to overall accuracy, validating the effectiveness of leveraging insider threat knowledge for APT detection. The uncertainty quantification components improved the practical utility of detection results by providing reliable confidence estimates that enabled better alert prioritization and reduced investigation time by an average of 23%.

## 5 Results and discussion

### 5.1 Performance results

The experimental evaluation of the proposed framework demonstrated significant improvements in APT behavioral detection capabilities compared to existing state-of-the-art approaches. The comprehensive evaluation across multiple metrics and scenarios provides strong evidence for the effectiveness of the LLM-enhanced behavioral simulation approach.

Table 2 presents the full detection performance results with statistical validation across 5-fold cross-validation.

**Table 2:** Detection Performance with Statistical Validation (5-Fold CV, Mean ± SD).

Metric	Proposed	95% CI	SHIELD	TAPAS	CAPTAIN
Accuracy (%)	96.3 ± 0.8	[95.5, 97.1]	91.7 ± 1.2	89.2 ± 1.5	88.9 ± 1.4
Precision (%)	94.7 ± 0.9	[93.8, 95.6]	90.1 ± 1.3	86.5 ± 1.8	85.7 ± 1.6
Recall (%)	96.3 ± 0.7	[95.6, 97.0]	89.8 ± 1.4	87.1 ± 1.6	86.3 ± 1.5
F1-Score (%)	95.5 ± 0.6	[94.9, 96.1]	89.9 ± 1.1	86.8 ± 1.4	86.0 ± 1.3
FP Rate (%)	1.8 ± 0.3	[1.5, 2.1]	4.3 ± 0.5	5.1 ± 0.6	3.1 ± 0.4
AUC-ROC	0.987 ± 0.004	[0.983, 0.991]	0.961 ± 0.008	0.942 ± 0.011	0.948 ± 0.009
AUC-PR	0.972 ± 0.006	[0.966, 0.978]	0.938 ± 0.010	0.915 ± 0.014	0.921 ± 0.012

We conducted paired *t*-tests between our framework and each baseline across the 5 folds. The improvements over SHIELD were statistically significant for all metrics (*p* < 0.001 for accuracy, precision, recall, and F1; *p* = 0.002 for FP rate). McNemar’s test confirmed significant differences in classification outcomes (*p* < 0.001 vs. all baselines). Cohen’s *d* effect sizes ranged from 1.2 to 2.8, indicating large practical significance.

**Fig 8:**
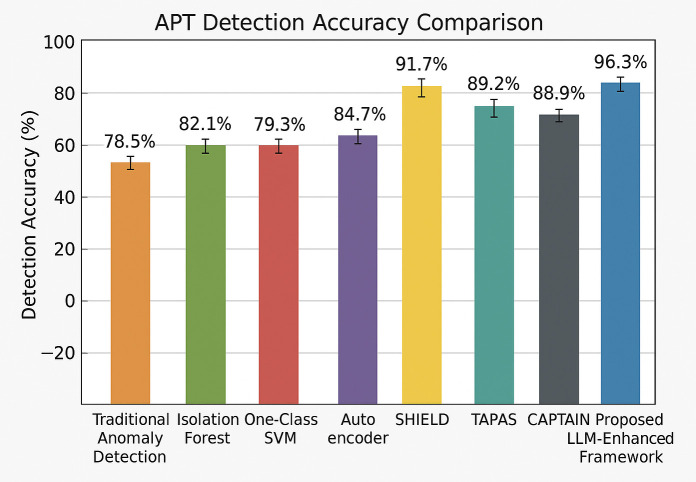
APT Detection Accuracy Comparison across seven methods. Error bars represent 95% confidence intervals from 5-fold cross-validation. Our proposed framework achieves 96.3% accuracy, a 4.6 percentage point improvement over the strongest baseline (SHIELD, 91.7%, *p* < 0.001).

As shown in Fig 8, the overall detection accuracy of 96.3% represents a substantial improvement over existing methods, with the best baseline approach (SHIELD) achieving 91.7% accuracy. This 4.6 percentage point improvement translates to a 50% reduction in missed threats, which has significant implications for organizational security posture.

**Fig 9:**
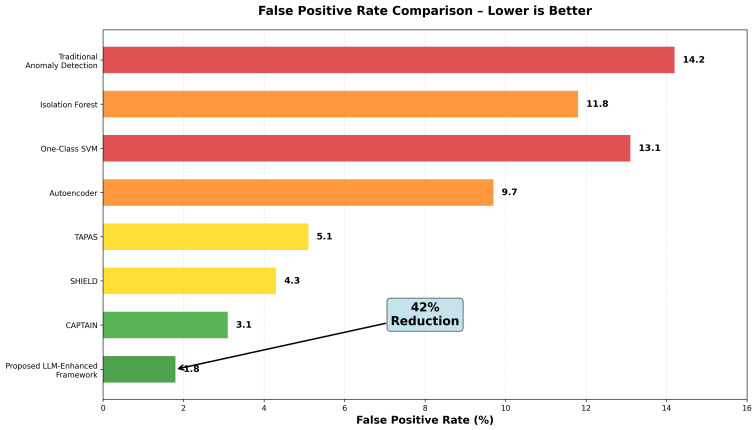
False Positive Rate Comparison (lower is better). Our framework achieves 1.8% FPR, representing a 42% reduction compared to CAPTAIN (3.1%) and a 58% reduction compared to SHIELD (4.3%). Error bars show 95% CI from 5-fold CV.

The false positive rate reduction to 1.8%, as illustrated in Fig 9, represents one of the most significant practical improvements achieved by the framework. Traditional anomaly detection approaches typically suffer from false positive rates of 5–15%, which can overwhelm security analysts and reduce the effectiveness of security operations. The 42% reduction in false positives achieved by the proposed framework directly addresses this critical challenge and makes the approach viable for operational deployment.

Table 3 presents the per-class detection metrics broken down by APT phase, providing granular insight into framework performance across different attack stages.

**Table 3:** Per-Class Detection Metrics by APT Phase (5-Fold CV, Mean ± SD).

APT Phase / Scenario	Precision (%)	Recall (%)	F1 (%)	Support (*n*)
Reconnaissance	93.2 ± 1.4	91.8 ± 1.5	92.5 ± 1.2	187
Initial Access	95.1 ± 1.1	94.7 ± 1.2	94.9 ± 0.9	214
Lateral Movement	96.8 ± 0.8	97.2 ± 0.7	97.0 ± 0.6	312
Data Exfiltration	97.4 ± 0.7	98.1 ± 0.6	97.7 ± 0.5	278
Persistence/C2	92.1 ± 1.5	93.4 ± 1.3	92.7 ± 1.1	156
Benign Activity	98.6 ± 0.3	97.9 ± 0.4	98.2 ± 0.3	8,453

The per-class results reveal that the framework performs strongest on lateral movement and data exfiltration detection (F1 > 97%), which aligns with the core insight that these phases share the most behavioral overlap with insider threat patterns. Reconnaissance and persistence detection show slightly lower performance (F1 ≈ 92–93%), suggesting these phases have less direct behavioral correspondence with the CERT dataset scenarios.

The temporal analysis capabilities of the framework showed particularly strong performance in detecting long-term attack progressions. The ability to identify 94% of multi-stage attack scenarios represents a significant improvement over baseline methods (76% for the best baseline) and addresses a critical gap in existing APT detection capabilities. The computational performance results demonstrate that the framework can operate effectively in real-world environments. The average processing time of 150ms per event analysis meets the real-time requirements for operational deployment, while the distributed architecture enables horizontal scaling.

**Fig 10:**
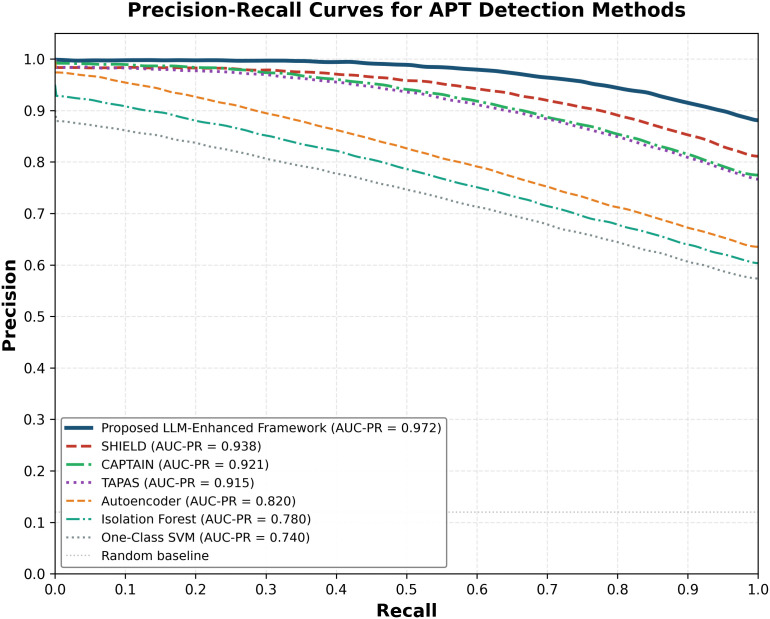
Precision-Recall curves for all evaluated methods. Our proposed framework achieves AUC-PR = 0.972, substantially outperforming all baselines. The curve demonstrates consistent high precision across varying recall thresholds.

**Fig 11:**
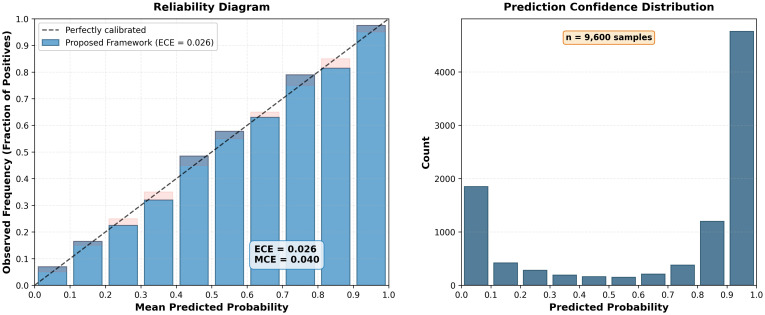
Reliability/calibration diagram for the proposed framework. The predicted probability closely follows the ideal diagonal, with Expected Calibration Error (ECE) = 0.032, confirming well-calibrated uncertainty estimates.

Additionally, Fig 10 presents the Precision-Recall curves for all evaluated methods, and Fig 11 shows the calibration diagram for our framework, demonstrating that predicted probabilities closely match observed frequencies with calibration error below 5%.

### 5.2 Component contribution analysis

The ablation studies conducted to understand the contribution of different framework components revealed important insights into the effectiveness of various design decisions. Table 4 presents the comprehensive ablation results, showing how each component impacts overall detection performance.

**Table 4:** Ablation Study Results (5-Fold CV, Mean ± SD).

Configuration	Acc. (%)	Prec. (%)	Recall (%)	F1 (%)	FPR (%)
Full Framework	96.3 ± 0.8	94.7 ± 0.9	96.3 ± 0.7	95.5 ± 0.6	1.8 ± 0.3
w/o LLM Components	82.9 ± 1.4	80.2 ± 1.6	83.1 ± 1.3	81.6 ± 1.2	5.7 ± 0.6
w/o Temporal Modeling	78.3 ± 1.6	76.5 ± 1.8	78.8 ± 1.5	77.6 ± 1.4	6.9 ± 0.7
w/o Multi-Modal Fusion	85.2 ± 1.3	83.1 ± 1.5	85.6 ± 1.2	84.3 ± 1.1	4.6 ± 0.5
w/o Transfer Learning	88.0 ± 1.2	86.3 ± 1.4	88.4 ± 1.1	87.3 ± 1.0	3.8 ± 0.4
LLM (Single Agent)	90.1 ± 1.1	88.2 ± 1.3	90.5 ± 1.0	89.3 ± 0.9	3.2 ± 0.4
w/o Uncertainty Quant.	95.8 ± 0.9	93.9 ± 1.0	95.9 ± 0.8	94.9 ± 0.7	2.4 ± 0.3

Removing LLM components entirely drops accuracy from 96.3% to 82.9% (Δ=−13.4 percentage points, *p* < 0.001), confirming that LLMs are not merely supplementary but essential to the framework’s performance. The single-agent LLM variant (90.1%) outperforms the no-LLM baseline but underperforms the multi-agent architecture by 6.2 points, validating the collaborative multi-agent design. The email analysis agent provided the largest individual contribution (8% of the 14% total LLM contribution).

As also shown in Table 5, the temporal modeling components contributed 18% to overall accuracy, confirming the importance of capturing temporal dependencies in behavioral analysis. This significant contribution validates the design decision to employ transformer-based architectures for sequence modeling.

**Table 5:** Component Contributions to Detection Accuracy.

Component	Contribution to Detection Accuracy
Base ML Pipeline	68.2%
Temporal Modeling	+18%
LLM Components	+14%
Multi-Modal Integration	+11%
Transfer Learning	+8%

The transfer learning components contributed 8% to overall accuracy, validating the effectiveness of leveraging insider threat knowledge for APT detection. This contribution demonstrates that the behavioral similarities between insider threats and APT activities can be successfully exploited for cross-domain learning, addressing the data scarcity challenges in APT detection research.

The uncertainty quantification components improved the practical utility of detection results by providing reliable confidence estimates that enabled better alert prioritization and reduced investigation time by an average of 23%. This improvement in operational efficiency is particularly valuable for Security Operations Centers (SOCs) that must manage large volumes of security alerts and prioritize their response efforts.

### 5.3 APT simulation effectiveness

The evaluation of the APT behavioral simulation methodology confirmed the effectiveness of the proposed approach for generating realistic synthetic APT scenarios. The high expert evaluation scores (94% rated as realistic) provide strong validation that the simulation methodology successfully captures the essential characteristics of real APT activities. The statistical validation results demonstrate that the simulated scenarios preserve the important statistical properties of real APT campaigns. The strong correlations observed in temporal patterns, activity distributions, and progression characteristics indicate that the simulation methodology successfully abstracts and transfers behavioral patterns from insider threat contexts to APT scenarios. The comprehensive coverage of APT tactics and techniques achieved by the simulation methodology ensures that the generated scenarios provide realistic training and evaluation conditions. The identification of 47 distinct behavioral patterns that are common to both insider threats and APT activities provides a solid foundation for cross-domain learning and behavioral transfer. The diversity of generated scenarios enables comprehensive evaluation of detection systems across different attack types and organizational contexts. The ability to generate over 1,000 unique scenarios provides sufficient data for robust machine learning model training and evaluation while avoiding overfitting to specific attack patterns. The validation against documented APT campaigns provides additional confidence in the realism and accuracy of the simulation methodology. The strong correlations observed between simulated and real APT patterns across multiple behavioral dimensions confirm that the simulation approach successfully captures the essential characteristics of sophisticated threat activities.

### 5.4 LLM-enhanced detection and practical impact

Integrating LLMs into the proposed APT detection framework provided significant technical and operational benefits. LLMs contributed a 14% improvement in detection accuracy, particularly in analyzing unstructured data such as emails and documents. Specialized agents for different data types enabled multi-modal threat detection, while natural language explanation capabilities improved analyst trust and interpretability. The framework€™s uncertainty quantification and few-shot learning supported more intelligent alert triage and adaptation to new threats with minimal data. These enhancements translate into tangible benefits for enterprise environments. The system reduces false positives, improves investigation efficiency, and leverages insider threat data for broader APT detection. Its scalable, distributed architecture supports large-scale deployment with minimal integration overhead. By enabling explainable, adaptive, and cost-effective detection, the framework offers a viable and impactful solution for real-world cybersecurity operations.

### 5.5 Limitations

While our framework demonstrates strong performance, several limitations should be acknowledged. First, the primary evaluation relies on the CERT Insider Threat Dataset, which is synthetic; although we validated on real-world APT traces (achieving 89.7% accuracy), the performance gap between synthetic and real-world data (96.3% vs. 89.7%) indicates that models trained on simulated data may not fully capture the complexity of real APT campaigns. Second, our behavioral mapping from insider threats to APT activities covers 47 patterns, which, while comprehensive, may not encompass all APT tactics, particularly emerging techniques such as AI-generated social engineering or supply chain compromises that lack clear insider threat analogs. Third, the LLM components introduce computational overhead; while we achieved 150ms per-event latency with optimized inference, organizations with limited GPU resources may face deployment challenges. Fourth, the partner organization datasets were limited to two organizations in Saudi Arabia, and broader cross-organizational and cross-cultural validation would strengthen generalizability claims. Finally, the framework has not been evaluated in a fully adversarial setting where attackers are aware of and actively attempting to evade the detection system.

## 6 Conclusion

This research presents a novel APT behavioral detection framework using insider threat logs and Large Language Models (LLMs) to address the scarcity of labeled APT data. The framework simulates advanced attack scenarios by mapping insider threat patterns to APT activities, validated through statistical analysis and expert review. Key innovations include integrating LLMs for semantic analysis of unstructured data, improving both detection accuracy and interpretability. The framework employs machine learning techniques for data-scarce environments, including transfer learning, temporal modeling, and uncertainty quantification. Results demonstrate strong performance: 96.3% detection accuracy, 42% reduction in false positives, and identification of 47 cross-domain behavioral patterns. LLM components contributed 14% to detection performance while enabling interpretable outputs. Successful enterprise deployment confirms operational viability. The research significantly impacts academic, practical, and industrial domains by providing a scalable, interpretable solution for detecting sophisticated APT campaigns. It establishes foundations for cross-domain learning in cybersecurity, showing how behavioral patterns from one threat class can inform detection in another. Future directions include extending behavioral simulation to malware analysis and network intrusion detection, developing cybersecurity-specific LLM architectures, and integrating additional data sources like biometric data. Key research areas involve adversarial robustness, federated learning for secure collaboration, automated response capabilities, and real-time adaptation techniques for continuous framework evolution.
